# Whole-Exome Sequencing in a South American Cohort Links ALDH1A3, FOXN1 and Retinoic Acid Regulation Pathways to Autism Spectrum Disorders

**DOI:** 10.1371/journal.pone.0135927

**Published:** 2015-09-09

**Authors:** Oscar A. Moreno-Ramos, Ana María Olivares, Neena B. Haider, Liga Colombiana de Autismo, María Claudia Lattig

**Affiliations:** 1 Departamento de Ciencias Biológicas, Facultad de Ciencias, Universidad de los Andes, Bogotá D.C., Colombia; 2 Schepens Eye Research Institute, Massachusetts Eye and Ear Infirmary, Department of Ophthalmology, Harvard Medical School, Boston, MA, United States of America; 3 Liga Colombiana de Autismo (LICA), Bogotá D.C., Colombia; Laboratoire de Biologie du Développement de Villefranche-sur-Mer, FRANCE

## Abstract

Autism spectrum disorders (ASDs) are a range of complex neurodevelopmental conditions principally characterized by dysfunctions linked to mental development. Previous studies have shown that there are more than 1000 genes likely involved in ASD, expressed mainly in brain and highly interconnected among them. We applied whole exome sequencing in Colombian—South American trios. Two missense novel SNVs were found in the same child: *ALDH1A3* (RefSeq NM_000693: c.1514T>C (p.I505T)) and *FOXN1* (RefSeq NM_003593: c.146C>T (p.S49L)). Gene expression studies reveal that Aldh1a3 and Foxn1 are expressed in ~E13.5 mouse embryonic brain, as well as in adult piriform cortex (PC; ~P30). Conserved Retinoic Acid Response Elements (RAREs) upstream of human ALDH1A3 and FOXN1 and in mouse Aldh1a3 and Foxn1 genes were revealed using bioinformatic approximation. Chromatin immunoprecipitation (ChIP) assay using Retinoid Acid Receptor B (Rarb) as the immunoprecipitation target suggests RA regulation of Aldh1a3 and Foxn1 in mice. Our results frame a possible link of RA regulation in brain to ASD etiology, and a feasible non-additive effect of two apparently unrelated variants in *ALDH1A3* and *FOXN1* recognizing that every result given by next generation sequencing should be cautiously analyzed, as it might be an incidental finding.

## Introduction

Autism spectrum disorders (ASD) are a range of complex neurodevelopmental conditions characterized by dysfunctions in mental development [1]. At the same time, it has been hypothesized that ASD are a group of continuous disorders instead of a single discrete disorder [2], explaining why ASD patient symptomatology is so variable. Although prevalence of ASD has increased worldwide [3], in Colombia there are no official reports regarding its prevalence.

A recent study has shown that ASD heritability is calculated closely to 50% [4] (lower than the ~90% previously estimated [2,5]) leading to higher environmental factor’s impact on the occurrence of the disorder. In fact, no clear environmental factors that have been unfolded since the recurrence of ASD does not change statistically between dizygotic twins and complete siblings [4].

Most of our understanding of the molecular basis of ASD comes from reports of rare copy number variants (CNVs) [6–8] and the discovery of *de novo* single nucleotide variants (SNVs) using Whole Exome Sequencing (WES) [9–13] or Whole Genome Sequencing (WGS) [14]. More than a 1000 genes are thought to be involved in ASD [15], most of them located in genes of known function and involved in pathways related to cell and neuronal development, projection, motility and proliferation; also in genes involved in spine and dendrite plasticity regulation, and gene regulation [8,15,16]. Additionally, variants affecting recurrent genes have been associated to ASD by their single impact ignoring a possible synergic impact between two or more de novo events in the same individual. Nonetheless, all this information has been derived mostly from Caucasian individuals. Thus, novel genes and even novel pathways involved in ASD pathogenesis might be found in understudied populations such as Colombian.

WES was applied in search of *de novo* variants that might be causative of ASD in four family trios from Colombia. Two *de novo* non-synonymous mutations affecting *ALDH1A3* (RefSeq NM_000693, MIM:600463) and *FOXN1* (RefSeq NM_003593.2, MIM:600838) genes were uncovered in the same child (FAM07). Recently, retinoic acid (RA) pathways mediated by Retinoid Orphan Receptor Alpha (RORA) have been implicated in ASD [17]. Furthermore, homozygous *ALDH1A3* missense and nonsense mutations in humans have been linked to anophthalmia and microphthalmia (MIM: 615113; A/M) with some affected individuals also exhibiting autistic traits [18–23]. Though no clear evidence supports a link between variants in ALDH1A3 with autism, mouse studies show that lack of Aldh1a3 (Gene NC_000073.6) results in abnormal GABAergic neuronal differentiation in the forebrain basal ganglia [24]. Malfunctions in this inhibitory system have been found to be associated to mental disorders like ASD, schizophrenia and bipolar disorder [25,26]. Moreover, failures of this enzyme in the striatum are related to elimination of dopamine receptor D2 in the nucleus accumbens [27].

Although little is known about *FOXN1*, it is essential for proper immune system function, especially in thymus development and maintenance during adulthood [28–30]. Foxn1 (Gene NC_000077.6) knockout mice (commonly known as NUDE mouse) overexpress pro-inflammatory cytokines (Specially T_H1_) [31]. This overexpression has also been reported in ASD children [32]. Besides T-cell immunodeficiency, mutations in FOXN1 have also been observed together with congenital alopecia and nail dystrophy (MIM: 601705). A homozygote mutation in FOXN1 was reported in a 15-week-old fetus with anencephaly and severe neural tube defect (MIM: 601705) [33].

As ALDH1A3 is an enzyme responsible for Retinoic Acid (RA) synthesis, and FOXN1 has been found to regulated by RORA (gene involved in RA cascade) [17], together with RA’s major role in gene expression regulation during brain development [34], it was questioned if RA might possibly regulate ALDH1A3 and FOXN1 through RA receptors (RARs). Predicted RA Response Elements (RAREs) in the promoter regions of both genes where evaluated to be recognized by RARs, using C57BL/6J (B6) mice, in two developmental stages. Rarb (Gene NC_000080.6) was selected as the chromatin association target protein since it is known to regulate dorsal and ventral telencephalon development, hippocampal plasticity and brain barrier development [35–38].

Results indicate that Rarb regulates Aldh1a3 and Foxn1 (NC_000077.6) in adult (~P30) mice PC and in embryo (~E13.5) whole brain, as demonstrated by ChIP essay. We suspect that both variants in genes ALDH1A3 and FOXN1 interact in a non-additive manner, where an epistatic interaction effect can be expected, possibly explaining the etiology in the affected child.

## Methods

### Ethics statement

The study presented here involves human participation and informed consent and assent. The study and consent procedure was approved by the ethical committee of Universidad de los Andes (Acta 11, 2011). Informed consent and assent was given and explained to all participants. All participants provided a written informed consent to participate in this study. Each consent was marked with a family and individual unique identifier, names and identifying information are kept securely stored. Only the principal investigator is able to recognize personal data with unique identifiers.

Animal procedures were approved by Schepens Eye Research Institute Animal Care and Use Committee (Permit number: S-309–0714) in compliance with the Animal Welfare Act Regulations.

### Cohort selection

Four family trios (family ID: Fam02, Fam07, Fam09 and Fam10) referred by Liga Colombiana de Autismo (LICA) were selected to participate in the study. All individuals are from Bogotá D.C.—Colombia. Affected individuals were engaged in a series of tests-tasks to evaluate patterns and maturity of the child, interaction with parents and family patterns. Inclusion in the present study required meeting criteria for autism on the ADOS [39] and ADI-R [40] and subject were confirmed to meet DSM-V (APA, 2012) criteria for autism by trained professionals who observed and interacted with them over several visits. After psychological examination, a genetic counselor physician discarded any comorbidity with other ASD related syndrome.

### DNA sample preparation, exome sequencing and analysis

DNA extraction was carried out using FlexiGene DNA kit (Qiagen, Gaithersburg, MD, USA). The extraction was carried out parting from 300uL of leucocytes following manufacturer instructions. A mean of 8.7ug of DNA per individual was sent to Otogenetics Corporation in Atlanta GA, USA. We applied WES using Agilent Sureselect All Exon V4 platform and PE100 Illumina HiSeq2000 platform to the four family trios selected (all family trios DNA samples met the quality criteria for WES by Otogenetics Corporation, Atlanta, GA—USA). Sequence reads were cleaned using the latest version of FASTX-Toolkit (V 0.0.13.2) (http://hannonlab.cshl.edu/fastx_toolkit/index.html). Sequence mapping and variant calling was carried first by mapping the short sequences using Burrows-Wheeler aligner (BWA) (from http://bio-bwa.sourceforge.net) [41] on the latest version of the human genome available at UCSC Genome Browser (HG19). SAM tools (from http://samtools.sourceforge.net) [42] and PicardTools (from http://picard.sourceforge.net) were used to manipulate and mark sequence duplicates. SNPs and small indel calling was achieved using HaplotypeCaller walker of Genome Analysis Toolkit (GATK, from http://www.broadinstitute.org/gatk/) [43,44]. Best practices of GATK were followed in order to guarantee an efficient variant calling. Annotation was performed using snpEff (Human genome reference 37.5) [45]. Putative *de novo* variants were identified using AWK language.

### Variant prioritization and validation

After identifying all the non-inherited variants, minor allele frequency (MAF) was revised from dbSNP. Those variants that presented a MAF > 5% were discarded. Different sets of primers were manually designed to flank the variants position obtained during calling. PCR amplification protocol described in Perea, C.S. et al., 2012 was used [46]. Sanger sequencing at Macrogen, Seul—Korea; and at Universidad de los Andes’ Sanger sequencing facility (3500 Genetic Analyzer; Applied Biosystems, USA) was used to corroborate the presence of each of the variants unveiled.

After validation of each of the variants was performed, depending on the predicted impact, different bioinformatic tools were used to predict their impact. The possible effect of the synonymous variant on mRNA stability was analyzed using SilVA [47], and ESEfinder for possible *de novo* splicing regions [48,49]. Non-synonymous variants were analyzed using SIFT [50], PolyPhen [51] and PROVEAN [52].

### Retinoic acid regulation pattern discovery

Searching for the relaxed Retinoic Acid Response Element [RARE; 5’-(A/G)G(G/T)(G/T)(G/C)A-3’ motif separated by one, two or five nucleotides, which interacts with heterodimers of RARs and retinoid X receptors (RXRs)] [34,53,54], was achieved using the online interface of Patser (online site: http://stormo.wustl.edu/consensus/html/Html/main.html) [55]. Genomic region spanning 30000bp upstream to 1000bp downstream from the initial transcription site for humans and mice (Human genome build NCBI 37.5 and Mouse genome build GRCm38.p2) in both negative and positive strands was analyzed.

### Animals

Pregnant females and adults were obtained under standard conditions from Jackson Laboratory and housed in vivariums at Schepens Eye Research Institute. The study was carried out in strict accordance with the recommendations in the Guide for the Care and Use of Laboratory Animals of the National Institute of Health. Tissues were collected from C57BL/6J (B6) postnatal (~P30) and from B6 embryonic (~E13.5).

### Aldh1a3 and Foxn1 expression study

Transcription was observed after total RNA extraction from piriform cortex (PC) from both lobules of adult C57BL/6J (B6) brain mice (~P30), and B6 embryonic whole brain (~E13.5) using Trizol. PC was chosen since Aldh1a3 expression has been reported in this region in postnatal stages [56]. A total of 2μg of total RNA was reversed using random decamers, in independent biological triplicates using Retroscript (Ambion). PCR, using primers flanking the conserved splice regions of both genes (S2 Table), was used to check Foxn1 and Aldh1a3 transcription. PCR products were run on agarose (2%) ethidium bromide stained gel.

### Chromatin immunoprecipitation

Chromatin immunoprecipitacion assay (ChIP) against RARs-RARE interaction was used to validate the bioinformatic predictions. ChIP, followed by PCR against the interaction of Rarb with the predicted RAREs, was performed as described in Haider, N.B. et al., 2009 with modifications [57]. Briefly, three adult (~P30) mice PC and three embryo (~E13.5) mouse whole brain were collected. Each sample was sonicated using three cycles. First cycle: 1s pulse*1s wait between pulses; total of 20 pulses. Second cycle: 19 steps of ten pulses, 1s pulse*1s wait between pulses, 30 seconds wait between steps; total of 190 pulses. Third cycle: 10s pulse*20s wait between pulses; total of three pulses. Average DNA fragment size obtained was ~300bp. Aliquots for Input (Positive control), Rarb antibody (SC-552, Santa Cruz Biotechnology, Inc.) immunoprecipitation and rabbit IgG (2027, Santa Cruz Biotechnology, Inc.) (Negative control) were taken and incubated overnight. PCR was performed using primers flanking each RARE sequence found in mice (S3 Table), to amplify approximately 200 base pair amplicon. PCR conditions used are previously described in Haider, N.B. et al., 2001 where an annealing temperature of 58°C and 35 cycles were applied, and a dilution of 1:10 for the Input sample was used [58]. PCR products were visualized by ethidium bromide staining.

## Results

### Exome sequencing, technical outcomes, variant calling and *de novo* variant discovery

After applying WES, a ~69.1X coverage was yielded. In average, ~228,116.1 variants per family trio were obtained. A total of three SNVs and one Indel were validated by PCR amplification followed by Sanger sequencing (Table 1). We observed a transvertion-transition ratio of 1:2 and non-coding SNV or Indel were found in two of the families studied. None of the genes found in this study have been reported in the 982 WES studies [9–13], or in any of the ten WGS family trios [14] performed to date. All other NGS data is stored at the Universidad de los Andes bioinformatic facility and it is freely available upon request.

**Table 1:** Non-inherited variants found in families Fam07 y Fam10.

Family ID	Gene name	Chr.	Chr. Pos.	Ref. Allele	Altern. Allele	A.A. Change	Sanger Validation
Fam07	ALDH1A3	15	101454953	T	C	I505T	+
	FOXN1	17	26851543	C	T	S49L	+
Fam10	NR4A2	2	157186342	C	A	S119	+
	GAS8-AS1	16	90095617	G	GCTGCGGGGCAGC	·	+

### Variant prioritization and analysis

One synonymous *de novo* SNV, located in *NR4A2* (Table 1, RefSeq NM_006186.3, MIM:601828; c.779G>T [p.S119]) and one *de novo* insertion in the ORF GAS8-AS1 (Table 1, RefSeq NC_000016.10, MIM:605179; g.90095617_90095618insCTGCGGGGCAGC) were found in the proband of Fam10. The possible effect of the synonymous variant on mRNA stability was analyzed using SilVA [47], as in O’Roak, B.J. et al., 2012 another synonymous variant was reported in this gene [10], and ESEfinder for possible *de novo* splicing regions [48,49], but no effects on mRNA stability or possible new splice sites were found (Table 2). The insertion in GAS8-AS1 (Table 1) was analyzed using ESEfinder to determine if it might alter *GAS8* gene (MIM:605178) splice since it is located in intron 2. ESEfinder did not show any new probable splicing site for the Indel in GAS8-AS1 or even *GAS8* (Table 2).

**Table 2:** Predicted impact using SIFT, PROVEAN and PolyPhen to the non-synonymous *de novo* Novel variants in proband from family Fam07.

Family	Variant class	Gene	Software				
			SilVA	ESEfinder	PROVEAN (Cutoff<-2.5)	SIFT (Cutoff<0.05)	PolyPhen (Cutoff<0.878)
FAM07	Non-Synonymous	ALDH1A3	NA	NA	Deleterious (-3.534)	Tolerated (0.142)	Possibly Damaging (0.878)
		FOXN1	NA	NA	Neutral (-0.241)	Harmful (0.006)	Benign (0.137)
FAM10	Synonymous	NR4A2	Likely benign	No splice site found	NA	NA	NA
	Insertion	GAS8-AS1	NA	No splice site found	NA	NA	NA

**Fig 1:**
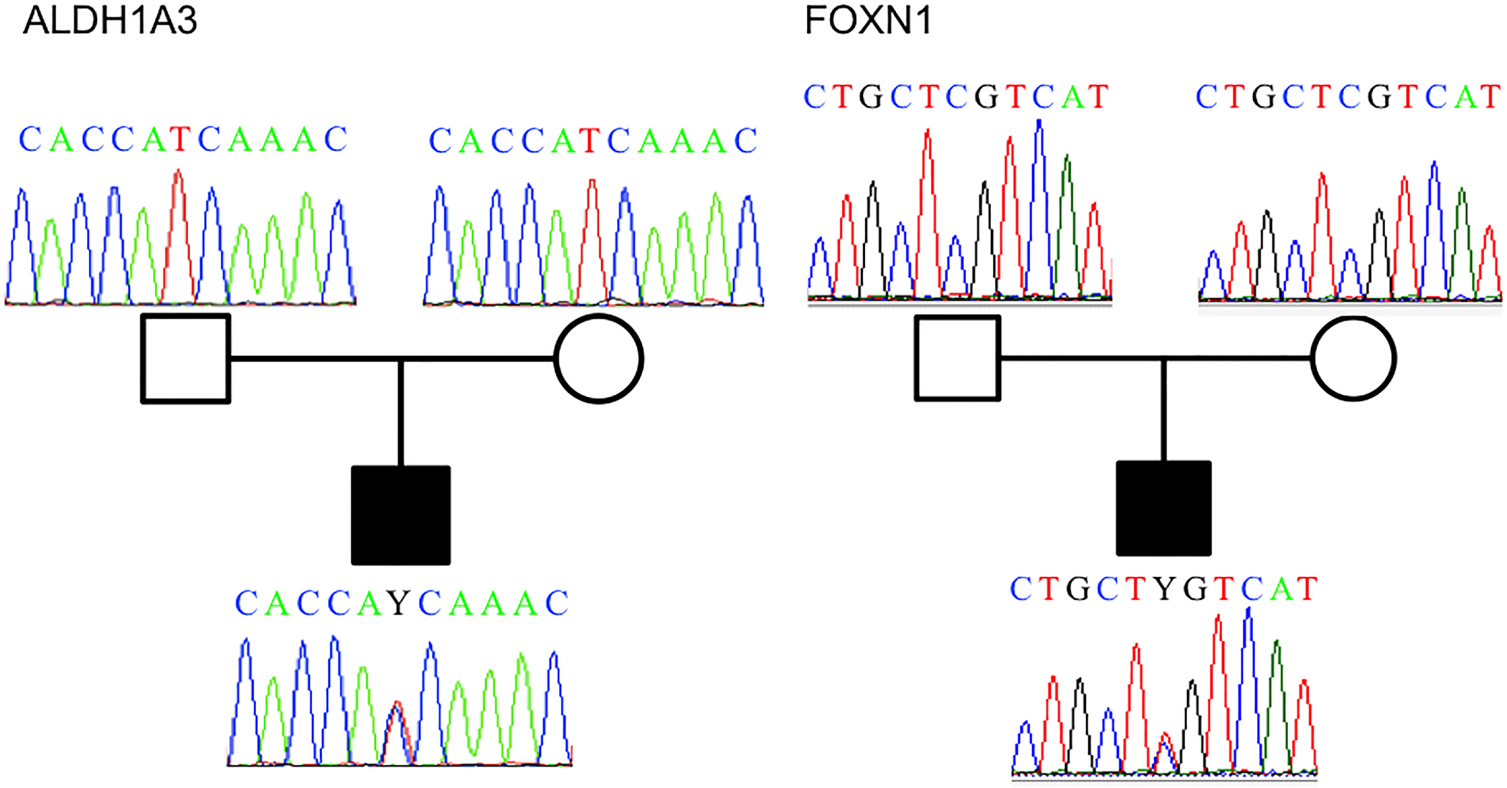
Pedigrees of family FAM07 showing chromatograms where the de novo Novel mutation event occurred for genes ALDH1A3 (c.1618T>C [p.Ile505Thr]) and FOXN1 (c.175C>T [p.Ser49Leu]).

Two non-synonymous *de novo* variants were uncovered in the affected child of family Fam07 within *ADLH1A3* (RefSeq NM_000693: c.1514T>C (p.I505T)) and *FOXN1* (RefSeq NM_003593: c.146C>T (p.S49L)) (Fig 1). SIFT [50] predictions indicate that the *FOXN1* alteration is harmful; while PolyPhen [51] and PROVEAN [52] predict that the SNV located in *ADLH1A3* is deleterious (Table 2). The other two families: Fam02 and Fam09 did not reveal any de novo mutations on the affected probands. The study further focused on studying both *ALDH1A3* and *FOXN1* since the variants found have a potential negative impact.

### RAREs prediction

After looking for the RAREs patter using Patser in the positive and negative strands of the promoter region of genes ALDH1A3 and FOXN1 in humans, as in Aldh1a3 and Foxn1 in mice [55], four possible RAREs for *ALDH1A3* and 18 for *FOXN1* in humans, and ten possible RAREs for Aldh1a3 and 11 for Foxn1 in mice were found (S1 Table).

### Aldh1a3 and Foxn1 expression in mice brain tissues and regulation by Rarb

**Fig 2:**
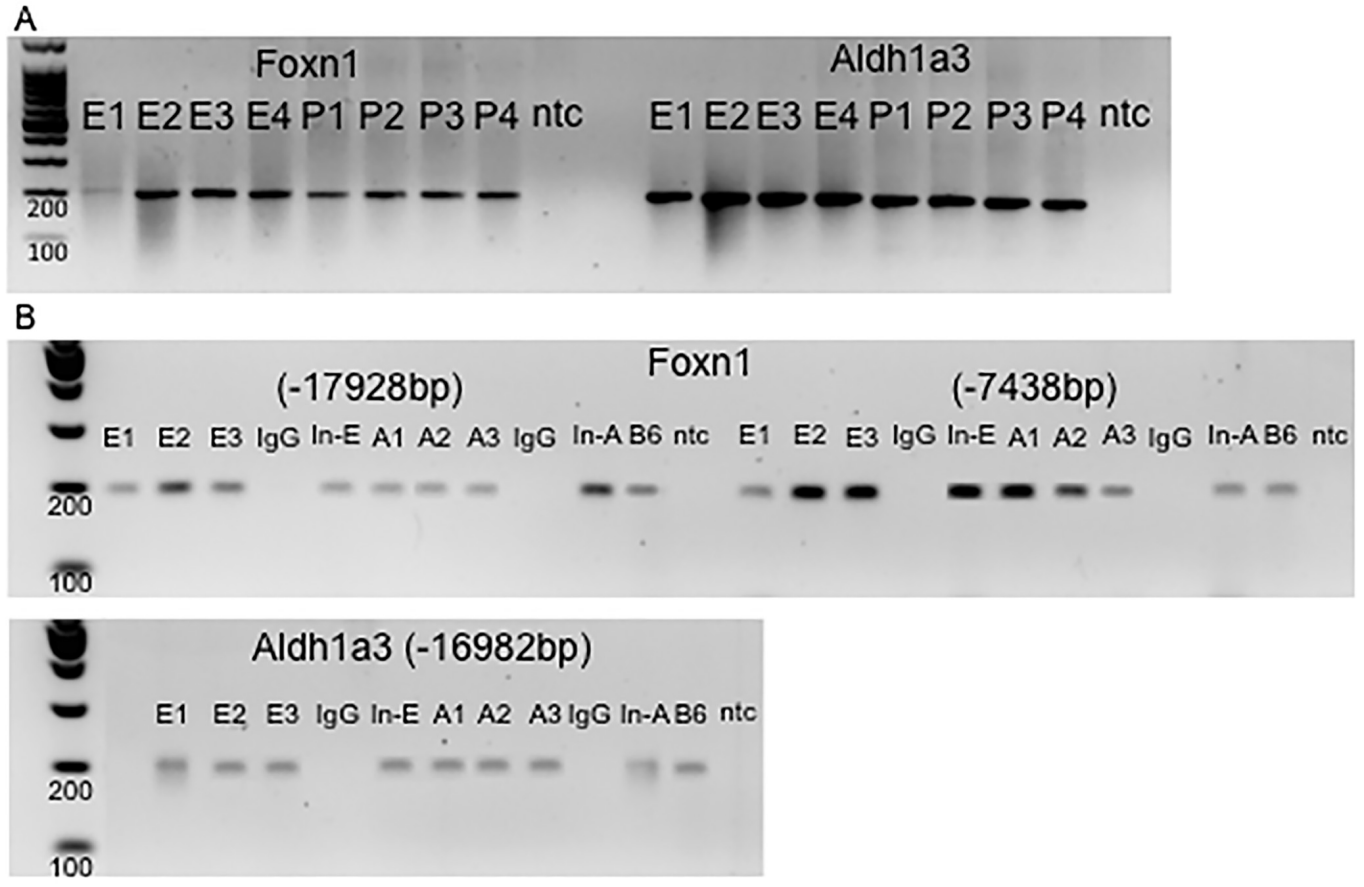
Transcription and ChIP PCR results for genes Foxn1 and Aldh1a3 in adult ~P30 (A) piriform cortex and Embryo ~E13.5 (E) mice. **A)** Foxn1 and Aldh1a3 message is observed at ~E13.5 and ~P30 stages. **B)** Rarb-Rare interaction observed for two predicted RAREs (-17828bp and-7438bp upstream initial transcription site) in gene Foxn1 and one in gene Aldh1a3 (-16982 upstream initial transcription site). In-E: Input control for embryo, In-A: Input control for adult.

According to the expression analysis performed on adult (~P30) PC and in embryo (~E13.5) whole brain, electrophoretic run indicates that both genes are transcribed in ~P30 mouse PC and in ~E13.5 mouse whole brain Fig 2A.

Chromatin Immunoprecipitation results demonstrate that Rarb binds to one predicted RARE upstream of Aldh1a3 and to two predicted RAREs upstream of Foxn1 in the adult PC (Fig 2B, Table 3) in mice. In the embryo’s stage, we observed that Rarb binds to one predicted RARE for Aldh1a3 and two for Foxn1 (Fig 2B, Table 3) in mice. These results indicate that both RAREs are probably recognized independently of the developmental stage.

**Table 3:** RARE sequences and their relative positions to the initial transcription site, found by ChIP for genes Foxn1 and Aldh1a3 in mice in adult (~P30) piriform cortex and embryo (~E13.5) whole brain.

Gene	RARE Sequence	Relative Position	Strand
Aldh1a3	GGGGGAGTGGGGGA	-16982	-
Foxn1	AGGTGACAATGGGGTGA	-7422	+
	GGTTCATCAGTTCA	-17785	+

## Discussion

Most ASD studies highlight a strong genetic heterogeneity where both *de novo* germline and rare inherited variants are distributed across numerous genes yet, interconnected in similar biological processes such as chromatin remodeling and transcription regulation [8,10–13,16] or target of fragile X mental retardation protein (FMRP) [59]. Furthermore, previous WES studies associate genes to ASD only if two or more disrupting variants are observed in the same gene in different individuals. Variant and gene association depends uniquely on the effect the affected gene has [6,10–13,15,59], though it is also important to examine how two or more de novo variants in the same individual might play a synergic role on ASD phenotype. Moreover, every result given by WES or WGS trio studies should be carefully analyzed, as it might be an incidental finding unless function studies are performed.

The findings of RAREs sequences in the promoter regions, in humans as in mice, made us consider that RARs family might regulate FOXN1 and ALDH1A3 genes. Thus, we aimed to determine if any of those RAREs predicted might be functional using mice as models. We first confirm expression of Aldh1a3 in PC during in adult (~P30) mouse brain as previously shown [56] and evaluated co-expression of Foxn1 in the same region and stage. Moreover, we demonstrate that both genes are also expressed in embryo (~E13.5) mice. ChIP-PCR results suggest that Foxn1 and Aldh1a3 expression is promoted by Rarb binding to different RARES in both tissues (adult PC and embryo whole brain; Fig 2B, Table 3). Since ChIP was only carried out to analyze Rarb-RARE interaction, we could not assure that any other members of the Rar family or other transcription factors might contribute to Foxn1 or Aldh1a3 regulation during the stages analyzed. Therefore, it is possible that gene RA regulation is exerted by other proteins in both tissues at both stages. Based on known functions of RARs/RXRs, these results predict a possible positive regulation by Rarb for both genes. The RA regulation model predicts that in RA presence, RARs/RXRs release co-repressor proteins and recruit co-activation complexes [34]. In fact, it has been demonstrated that RA promotes Rars binding to putative RAREs in mouse embryonic stem cells working as transcription enhancers [60]. As Rarb and the other RAR family members regulate many genes at different stages and tissues at time-specific periods, lack of RA might contribute to a transcriptional deregulation.

Recently, non functional *ALDH1A3* alleles have been reported as responsible for ophthalmologic diseases as A/M in which four of the six reports mention that some affected individuals also have ASD like features or intellectual disability in different degrees, even though these findings are generally considered as incidental [18,19,21,23]. However, it is not possible to discard the idea that mutations in *ALDH1A3* might alter brain development since it has been described that RA is essential for brain cortex development facilitating transcription of essential genetic markers [61]. Additionally, FAM07 child carries a non-synonymous variant, predicted to be pathogenic in *ALDH1A3* (Table 2). One would thus predict that RA synthesis and concentration in basal ganglia, nucleus accumbens, striatum, and PC would be lower than normal levels.

Since RA mediates transcription of different genes, variants in ALDH1A3 in conjunction with other inherited rare variants or *de novo* variants potentially act synergistically causing ASD. This might also be the case in the A/M phenotype where some of the affected individuals also have ASD or intellectual disability [19]. Yet, if another pathogenic variant is present on a gene that is regulated by retinoic acid, the altered concentrations levels of RA together with a deficient function of a second gene might end up in a phenotype such as ASD.

As no clear data of FOXN1 regulation of brain development is clear, we cannot predict a direct effect of the non-synonymous variant observed in this child. Since this nuclear receptor is apparently regulated by RA via Rarb, the lack of this metabolite due to the mutation in *ALDH1A3* might cause a lower transcription of *FOXN1*, leading to a dysregulation of genes and pathways not yet described. Moreover, nude affected humans by mutations or truncations in the FOXN1 gene do not present gross central nervous system alterations [62], but they however present changes in the corpus callosum, neural tube and choroid plexus [33,62]. This suggest that *FOXN1* has similar functions as other member of the FOX family like *FOXP1* and *FOXP2* [63,64], which have been already associated to ASD [6].

In conclusion, the results indicate that next generation sequencing from under-studied cohorts provide new genetic data that might conduct to new perspectives and hypothesis to explain genetic basis of ASD. Our results are in concordance with previous studies demonstrating that transcription factors play significant roles in ASD [8,10–13,16]. A previous study of our group reported that genes involved in transcription regulation are essential to serotoninergic pathway stability in depression [65]. Moreover, even though ASD is highly heritable, a multitude of environmental influences play a fundamental role on ASD incidence [4], which might interact with the proband’s genetic variants causing the disorder. Though carrying a variant in one allele of ALDH1A3 might not cause a defined phenotype such as A/M, this can act as a risk allele to ASD since lower levels of RA might contribute to a poor transcription of several genes, one of them being FOXN1. Additionally, the second gene variant present in FOXN1, might affect this transcription factor´s function by not properly regulating yet uncovered pathways in brain. Finally, when attributing causation to *de novo* variants prudence needs to be exercised.

## Supporting Information

S1 Table Retinoic Acid Response Elements (RAREs) found 30000bp upstream and 1000bp downstream from the initial transcription site in both DNA strands for ALDH1A3 and FOXN1 genes in humans, and Aldh1a3 and Foxn1 genes in mice. Relative positions are given from the initial transcription site of each gene.
(PDF)

S2 Table Primer sequences flanking conserved regions of Aldh1a3 and Foxn1 transcripts in mice. Sequences are given 5’࢐3’ direction.
(PDF)

S3 Table Primer sequences used to amplify each flanking RARE bioinformatically predicted sequences for Aldh1a3 and Foxn1 genes in mice. Sequences are given 5’→3’ direction.
(PDF)
