# Supplementary material for: Whole-Exome Sequencing in a South American Cohort Links ALDH1A3, FOXN1 and Retinoic Acid Regulation Pathways to Autism Spectrum Disorders
Source: PLoS One. 2015 Sep 9;10(9):e0135927. doi: 10.1371/journal.pone.0135927 (PMC4564166; doi:10.1371/journal.pone.0135927)
Supplement: S1 Table — Relative positions are given from the initial transcription site of each gene. (PDF) [file pone.0135927.s001.pdf]

Supplemental data

Whole-Exome Sequencing in a South American Cohort Links ALDH1A3, FOXN1 and Retinoic Acid Regulation Pathways to Autism Spectrum Disorders

Oscar. A. Moreno-Ramos[1], Ana María Olivares[2], Neena B. Haider[2], Liga Colombiana de Autismo[3], María C. Lattig[1]

[1] Universidad de los Andes, Facultad de Ciencias, Departamento de Ciencias Biológicas, Bogotá D.C. – Colombia

[2] Schepens Eye Research Institute, Massachusetts Eye and Ear Infirmary, Department of Ophthalmology, Harvard Medical School, Boston (MA) – United State

[3] Liga Colombiana de Autismo, Bogotá D.C. – Colombia

Table S1. Retinoic Acid Response Elements (RAREs) found 30000bp upstream and 1000bp downstream from the initial transcription site in both DNA strands for ALDH1A3 and FOXN1 genes in humans, and Aldh1a3 and Foxn1 genes in mice. Relative positions are given from the initial transcription site of each gene.

Gene	RARE pattern	Relative Position	Strand	Score	ln(P-value) < CutOff
**Human**					
ALDH1A3	5'-(A/G)G(G/T)(G/T)(G/C)A-NN-(A/G)G(G/T)(G/T)(G/C)A-3'	-8744<br>-13955	-<br>+	10.61	-11.09 < -9.456
	5'-(A/G)G(G/T)(G/T)(G/C)A-N-(A/G)G(G/T)(G/T)(G/C)A-3'	-8815<br>-26896	-<br>-	10.58	-11.09 < -9.441
FOXN1	5'-(A/G)G(G/T)(G/T)(G/C)A-NNNNN-(A/G)G(G/T)(G/T)(G/C)A-3'	-4574<br>-12786<br>-21671<br>-23466<br>-27282<br>-28005	-<br>-<br>+<br>-<br>+<br>-	10.69	-11.09 < -9.489
	5'-(A/G)G(G/T)(G/T)(G/C)A-NN-(A/G)G(G/T)(G/T)(G/C)A-3'	-4153<br>-6261<br>-7716<br>-10156<br>-16969<br>-23267<br>-24194<br>-26456<br>-27258<br>-29727	+<br>-<br>-<br>+<br>-<br>+<br>+<br>+<br>+<br>-	10.61	-11.09 < -9.456
	5'-(A/G)G(G/T)(G/T)(G/C)A-N-(A/G)G(G/T)(G/T)(G/C)A-3'	-1112<br>-12480	+<br>+	10.58	-11.09 < -9.441
**Mouse**					
Aldh1a3	5'-(A/G)G(G/T)(G/T)(G/C)A-NNNNN-(A/G)G(G/T)(G/T)(G/C)A-3'	-1967<br>-24615	+<br>-	10.69	-11.09 < -9.489
	5'-(A/G)G(G/T)(G/T)(G/C)A-NN-(A/G)G(G/T)(G/T)(G/C)A-3'	-16982	-	10.61	-11.09 < -9.456
	5'-(A/G)G(G/T)(G/T)(G/C)A-N-(A/G)G(G/T)(G/T)(G/C)A-3'	-5675<br>-13311<br>-13642<br>-18348<br>-19711<br>-21905<br>-29711	+<br>-<br>-<br>-<br>-<br>-<br>-	10.58	-11.09 < -9.441
Foxn1	5'-(A/G)G(G/T)(G/T)(G/C)A-NNNNN-(A/G)G(G/T)(G/T)(G/C)A-3'	-7422<br>-8341<br>-8732<br>-10426<br>-21635	+<br>-<br>+<br>-<br>-	10.69	-11.09 < -9.489
	5'-(A/G)G(G/T)(G/T)(G/C)A-NN-(A/G)G(G/T)(G/T)(G/C)A-3'	-2934<br>-17785<br>-21624<br>-29571	+<br>+<br>-<br>-	10.61	-11.09 < -9.456
	5'-(A/G)G(G/T)(G/T)(G/C)A-N-(A/G)G(G/T)(G/T)(G/C)A-3'	-13060<br>-29307	-<br>-	10.58	-11.09 < -9.441
