# Supplementary material for: Whole-Exome Sequencing in a South American Cohort Links ALDH1A3, FOXN1 and Retinoic Acid Regulation Pathways to Autism Spectrum Disorders
Source: PLoS One. 2015 Sep 9;10(9):e0135927. doi: 10.1371/journal.pone.0135927 (PMC4564166; doi:10.1371/journal.pone.0135927)
Supplement: S2 Table — Sequences are given 5’࢐3’ direction. (PDF) [file pone.0135927.s002.pdf]

Supplemental data

Whole-Exome Sequencing in a South American Cohort Links ALDH1A3, FOXN1 and Retinoic Acid Regulation Pathways to Autism Spectrum Disorders

Oscar. A. Moreno-Ramos[1], Ana María Olivares[2], Neena B. Haider[2], Liga Colombiana de Autismo[3], María C. Lattig[1]

[1.] Universidad de los Andes, Facultad de Ciencias, Departamento de Ciencias Biológicas, Bogotá D.C. – Colombia
[2.] Schepens Eye Research Institute, Massachusetts Eye and Ear Infirmary, Department of Ophthalmology, Harvard Medical School, Boston (MA) – United State
[3.] Liga Colombiana de Autismo, Bogotá D.C. – Colombia

Table S2. Primer sequences flanking conserved regions of Aldh1a3 and Foxn1 transcripts in mice. Sequences are given 5'→3' direction.

Primer name	Primer Sequence
FOXN1_F	GGGGCAGCTGAGAGCTAAAA
FOXN1_R	TCCCTAAGGGGTTCACAGGT
ALDH1A3_F	CTGATGCTGGCCTGGAAACT
ALDH1A3_R	TGTTGATCTGCGGATGGGAG
