# Supplementary material for: Whole-Exome Sequencing in a South American Cohort Links ALDH1A3, FOXN1 and Retinoic Acid Regulation Pathways to Autism Spectrum Disorders
Source: PLoS One. 2015 Sep 9;10(9):e0135927. doi: 10.1371/journal.pone.0135927 (PMC4564166; doi:10.1371/journal.pone.0135927)
Supplement: S3 Table — Sequences are given 5’→3’ direction. (PDF) [file pone.0135927.s003.pdf]

Supplemental data

Whole-Exome Sequencing in a South American Cohort Links ALDH1A3, FOXN1 and Retinoic Acid Regulation Pathways to Autism Spectrum Disorders

Oscar. A. Moreno-Ramos[1], Ana María Olivares[2], Neena B. Haider[2], Liga Colombiana de Autismo[3], María C. Lattig[1]

[1.] Universidad de los Andes, Facultad de Ciencias, Departamento de Ciencias Biológicas, Bogotá D.C. – Colombia

[2.] Schepens Eye Research Institute, Massachusetts Eye and Ear Infirmary, Department of Ophthalmology, Harvard Medical School, Boston (MA) – United State

[3.] Liga Colombiana de Autismo, Bogotá D.C. – Colombia

Table S3. Primer sequences used to amplify each flanking RARE bioinformatically predicted sequences for Aldh1a3 and Foxn1 genes in mice. Sequences are given 5'→3' direction.

Gene	Position	Strand	Forward primer	Reverse primer
Aldh1a3	-1967	+	CCCTCTTACTGATTCCCATG	CCTGCTGAATGACAAGCTGA
	-5675	+	Region not suitable for primer design	Region not suitable for primer design
	-13311	-	CCAACATGGCTTCTTCATGGA	TCCCTCCTTCCCATGCTAACT
	-13642	-	TCCTAAGGGCAGAGTTGATCTCA	TGAAGAAGCCATGTTGGAAGAA
	-16982	-	CCCCTGGAGCTTGTGTCTCTA	ACATTTCCAATGCTATCCCAAAA
	-18348	-	TCCGGGAGTAACTTGCAACAT	CCTGAGGGCTAACTGCATGAC
	-19711	-	GCAGGGTTCAGTCTACCAGGAA	TTGGCAGGAGGCTTCAAAAT
	-21905	-	CTCCATGCCGGGATATTTGA	ATCTCTCCTCCCAAACAAACGA
	-24615	-	CTCTGCTGGGTTTCCTGTGAA	AGGACAGCCTTGGGTTCTCA
	-29711	-	TTCAAAGCATTGCCTTGTTGA	CAGCAATGACCTTTGACCTTATGA
Foxn1	-2934	+	AAAAAGATGTTGCATGGAAGCA	TGAAGCCTCTCTGGGAGTTCTG
	-7422	+	TGCTGGACGACCTGACTCTCT	CTGTCCAAGACACCTGCAAGAC
	-8341	-	CCTCAGCCTCTTGAGTGAAACC	CAACACCTGTATGGCCCAACT
	-8732	+	CAGGTAACCATAGTAGCAGAGAAGATTC	GGGTGAGGTGTGTCAGGAGAGT
	-10426	-	GTTCTACAGTACCAAGTCAC	CTGCAGTGTTAGGCAATGTC
	-13060	-	GAGTTTTGCAGAGAAGGCTGTCA	CCGCCTTCTTCCTTCCTTTT
	-17785	+	CTGGTGCGATTCAAACTCAAGA	TTTGTGCCTGGTACCCTCAGA
	-21624	-	GAAGGGAAGACTTGAGCAGA	AGTTGTTCCTTGAGGTCCCA
	-21635	-		
	-29307	-	GGAGCTAGCTGCCCTTAGTACAGA	CAGGCAGCACTGGCATTTG
	-29571	-	GTGCTGTGTGACAGACACCATTT	GAGGCCTGACTTGAGCTCTGTAC
